# A single amino acid mutation in norovirus NS4 promotes viral spread

**DOI:** 10.1128/jvi.00086-26

**Published:** 2026-04-27

**Authors:** Mridula Annaswamy Srinivas, Robert C. Orchard

**Affiliations:** 1 Department of Immunology, University of Texas Southwestern Medical Center 12334, Dallas, Texas, USA; 2 Department of Microbiology, University of Texas Southwestern Medical Center 12334, Dallas, Texas, USA; Emory University School of Medicine, Atlanta, Georgia, USA

**Keywords:** viral spread, evolution, norovirus

## Abstract

Viruses can rapidly adapt and evolve to new, unfavorable environments due to their decreased replication fidelity, large reproductive index, and short life cycle. Often, these adaptations that enable increased fitness in a new, specialized environment come with a trade-off of decreased fitness in a standard, general environment. Understanding the mechanistic basis for these fitness trade-offs has provided important insight into vaccine development, mechanism of action of antivirals, and function of viral proteins. Here, we sought to identify how a specialist murine norovirus (MNV) could be converted to a jack of all trades through a novel mutation without genetic reversion. Previously, we found that a mutation in MNV (NS6^F182C^) overcame restriction by host protein Trim7 but decreased the efficiency of viral polyprotein NS6-7 cleavage and resulted in attenuation of this virus. Here, we find that a single valine-to-isoleucine mutation in MNV non-structural protein NS4 (NS4^V11I^) is sufficient to rescue the attenuated replication of NS6^F182C^ over multiple cycles of replication. However, NS4^V11I^ did not affect the defective polyprotein cleavage; instead, the mutation facilitates faster viral spread *in vitro* independent of interferon signaling. The emergence of this mutation in NS4^V11I^ suggests an unappreciated connection between NS4 and NS6 during norovirus replication and provides a system to define the unknown role of norovirus NS4 during infection.

**IMPORTANCE:**

Viruses and hosts are involved in a continuous arms race for survival. Often, when viruses evolve to specialize in specific host environments, they lose their versatility and become specialists, only able to grow in one setting. This feature has been leveraged to create live-attenuated vaccines, identify the mechanism of action of antivirals, and uncover fundamental aspects of viral replication. Using murine norovirus as a model system, we aim to understand how a virus can adapt to overcome replication inefficiencies imposed by a previously acquired adaptive mutation. Here, we identify an unexpected connection between two murine norovirus non-structural proteins and uncover a role for the viral protein NS4 in viral spread. Taken together, these data provide new insight into viral evolution and the functions of norovirus proteins.

## INTRODUCTION

A fundamental question in virology is how viral fitness is tuned. As viruses evolve, they balance the selective pressures from host antagonism with constraints on the ability of viral proteins to perform their essential tasks. As viruses adapt to become more specialized in replicating in one environment, they frequently lose the ability to robustly replicate in other environments (1, 2). Investigating the molecular basis for these fitness trade-offs in natural populations of viruses has been challenging due to the large number of mutations that exist between natural isolates and the balancing of multiple fitness barriers. Experimental evolution of viruses via *in vitro* passaging enables strong selective pressure to produce viruses with an altered host niche. These systems have allowed for the discovery of antiviral targets, intrinsic barriers, inefficiencies to infection, and how viruses adapt to extreme environments (2). In many cases, an adaptive mutation comes with a fitness cost in the non-selective environment, a phenomenon described as antagonistic pleiotropy (3). For example, selection of a ribavirin-resistant poliovirus leads to a virus that can replicate significantly better in the presence of nucleoside analogs (4), but is significantly attenuated in mice compared to the wild-type virus (5, 6). Similarly, a Coxsackievirus B3 selected for increased replication speed was significantly attenuated in mice (7). One way to avoid antagonistic pleiotropy is to have variance in the environment. For example, vesicular stomatitis virus (VSV) passaged onto either HeLa or MDCK cells becomes less fit in the other cell line. However, alternative passaging of VSV on HeLa and MDCK cells enables fitness gains across hosts, demonstrating that not all adaptations come with trade-offs (8). While most studies reveal generalists emerging from heterogeneous or variable environments, it is not the only way to obtain a generalist (9, 10). The *Pseudomonas syringae* bacteriophage Φ6 displays limited host range on *Pseudomonas syringae* pathovars but can expand its host range to other pathovars in experimental evolution studies (11). While most of the mutations identified were costly to fitness in the original host, a few mutations had no decrease in fitness on the original host (11). Thus, while many adapted organisms suffer from antagonistic pleiotropy, there are generalists that do not incur a fitness defect, so-called no-cost or cost-free generalists (11, 12). Despite these observations, a knowledge gap persists in defining the molecular mechanisms of how mutations ameliorate antagonistic pleiotropy and how cost-free generalists emerge. Such an investigation would provide information on the genetic interactions between viral proteins, likely giving molecular insight into evolutionary trade-offs.

Murine norovirus (MNV) is a leading model system to understand human norovirus infections in a natural environment. Human norovirus is the preeminent cause of gastroenteritis worldwide but is understudied due to the lack of a small animal model and limited replication of human norovirus *in vitro*. Noroviruses are non-enveloped, positive-sense RNA viruses and are members of the Caliciviridae family. In a genome-wide screen for antiviral factors toward MNV, we determined that Trim7 expression induces a potent anti-MNV restriction phenotype (13). Trim7 binds and ubiquitinates the MNV non-structural protein NS6, which is the norovirus 3C-like protease (14, 15). Using an *in vitro* passaging approach, we determined that mutations that limit polyprotein processing of the NS6 and NS7 precursor (NS6-7) confer protection against Trim7 (14). Decreased processing of NS6-7 via introduction of a single point mutation (NS6^F182C^) provided a fitness advantage for MNV replication in Trim7-overexpressing cells (14). However, the replication of MNV NS6^F182C^ is severely attenuated in wild-type cells and in mouse models of disease (14). Despite the strong phenotype *in vitro*, Trim7-deficient mice had no differences in MNV replication or mounting an innate immune response to MNV (16).

Despite the lack of an *in vivo* phenotype, several lines of evidence suggest that the MNV-Trim7 system is an ideal experimental system to uncover fundamental aspects of norovirus biology and viral evolution. First, most viruses within the Caliciviridae family maintain their protease and polymerase (the equivalent of NS6 and NS7 in noroviruses) as a fused precursor, in contrast to noroviruses. It is unclear why these closely related viruses have different requirements for processing these proteins. Thus, investigating the consequences of a fused NS6-7 in MNV provides insight into Caliciviridae evolution. Second, our previously identified adapted virus, MNV NS6^F182C^, provides a robust system to study fitness trade-offs in different environments (e.g., presence or absence of Trim7 overexpression). Third, advances in host and viral genetics enable facile genetic manipulation of both host and virus to study these questions in viral evolution.

Here, we used a forward genetic screen to identify a single suppressor mutation that enables MNV to replicate robustly even with inefficient cleavage of NS6-7. Unexpectedly, this mutation occurs in the viral non-structural protein 4 (NS4), consisting of a valine-to-isoleucine substitution. The NS4^V11I^ mutation not only improved the replication of a virus containing the NS6^F182C^ mutation but also improved the replication of the wild-type MNV strain. NS4^V11I^ did not promote increased polyprotein processing but enhanced the spread of MNV independent of interferon (IFN) signaling through STAT1. In summary, our data point to a genetic connection between processing of NS6-7 and MNV spread while also shedding light on how antagonistic pleiotropy can be overcome in viral populations.

## RESULTS

### Viral evolution screen for attenuated MNV^CW3^ NS6^F182C^

**Fig 1:**
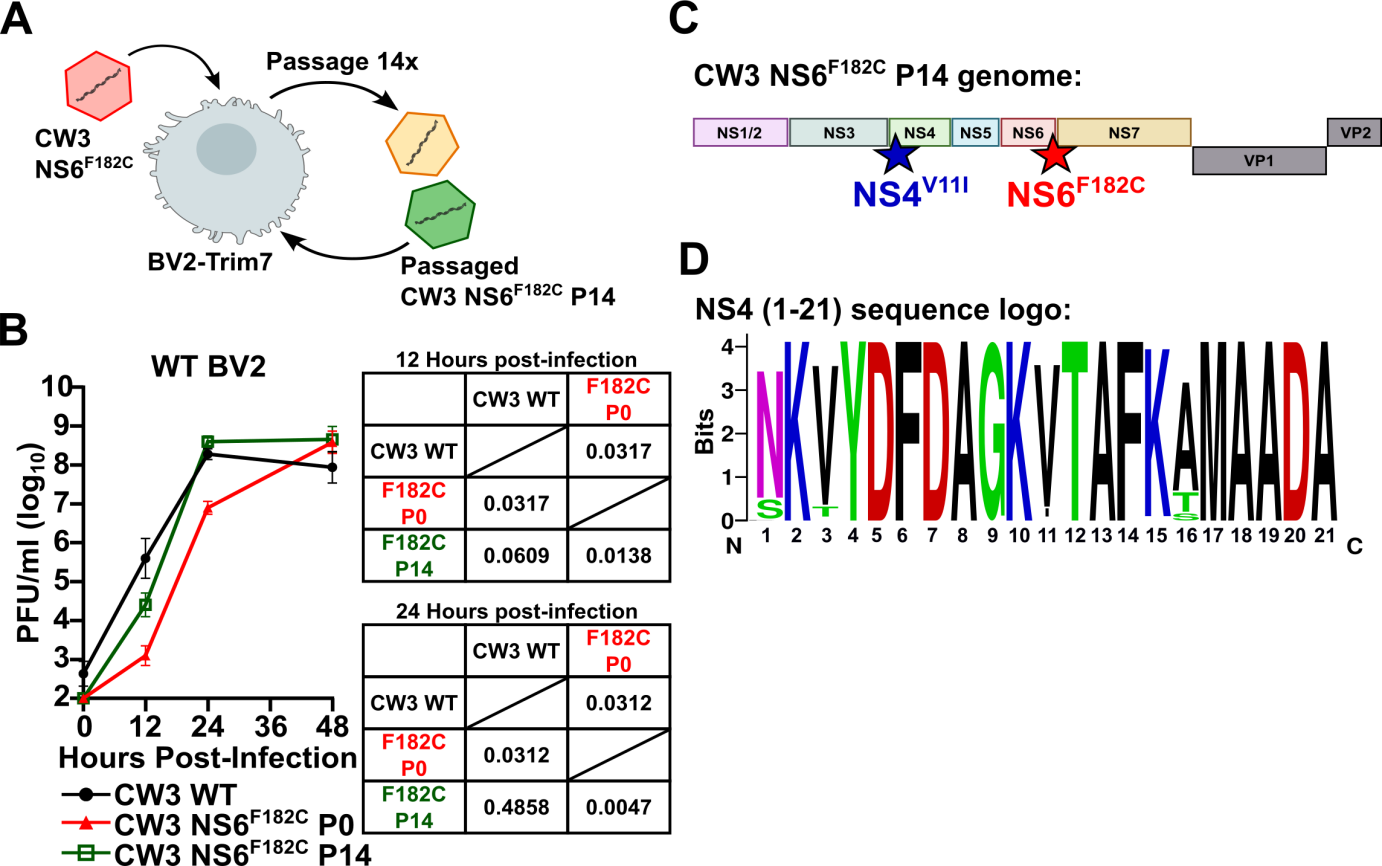
*In vitro* passaging of MNV^CW3^ NS6^F182C^ rescues its attenuated replication. (**A**) Cartoon schematic of the experimental strategy to rescue MNV strain CW3 NS6^F182C^ viral replication. BV2-Trim7 cells were infected with NS6^F182C^ virus and passaged initially every 48 h up to passage 6 (P6) and further passaged every 12 h up to P14. (**B**) Wild-type BV2 cells were infected with CW3 wild-type, parental CW3 NS6^F182C^ P0, or passaged CW3 NS6^F182C^ P14 at a multiplicity of infection (MOI) of 0.05. Viral replication was measured using plaque assays at indicated time points. Data are shown as mean ± SEM from three independent experiments. Tables show *P*-values of statistical comparisons of viral titers at 12 h and 24 h post-infection (HPI). Two-way ANOVA with Tukey’s multiple comparisons test. (**C**) Schematic of complete genome of CW3 NS6^F182C^ P14 representing mutations differing from CW3 wild type. NS6^F182C^ mutation was maintained, and an additional mutation in NS4 (NS4^V11I^) was discovered in this passaged virus. (**D**) Sequence logo of the first 21 amino acids of NS4 from 87 different MNV strains showing high conservation across all strains. Most MNV strains encode a valine at the 11th position, with some strains encoding an isoleucine, similar to the mutation found in the passaged CW3 NS6^F182C^ P14 virus.

We devised a genetic screen to regain the overall fitness of the MNV NS6^F182C^ while maintaining the Trim7 resistance phenotype. To do so, MNV strain CW3 harboring the NS6^F182C^ mutation (herein NS6^F182C^) was grown exclusively on Trim7-overexpressing BV2 cells (BV2-Trim7; Fig. 1A). We initially passaged the virus on BV2-Trim7 cells every 48 h for six passages but observed minimal rescue. To increase the selective pressure, we limited the time of viral replication to a single cycle of replication (12 h) for passages 7 through 14. The passage 14 (P14) virus had a significant increase in viral replication over the original MNV^CW3^ NS6^F182C^ (P0) in wild-type BV2 cells (Fig. 1B). Additionally, the replication of the P14 virus was similar to that of MNV^CW3^ wild-type (Fig. 1B).

To determine the genetic basis for this rescue in replication, we performed consensus sequencing of the P14 viral stock using RT-PCR and Sanger sequencing. Importantly, the NS6^F182C^ mutation was maintained in the P14 population; thus, we could rule out a genetic reversion for the restored replication (Fig. 1C). In addition to the F182C mutation, we identified an additional mutation in NS4 leading to a valine-to-isoleucine substitution (V11I; Fig. 1C). NS4 is highly conserved in all MNV strains, with most MNV strains encoding a valine at position 11, while some strains encode an isoleucine (Fig. 1D). The precise role of NS4 during norovirus replication is unknown, as it has no predicted enzymatic activity and no homology to functional domains. Interestingly, two prior investigations identified NS4^V11I^ emerging in viral stocks (17, 18). NS4^V11I^-containing viruses replicated slightly better *in vitro* than the parental virus, although no other phenotypes were attributed to this mutation (18). Thus, we have uncovered an unappreciated genetic connection between the NS6F182C Trim7-adaptive mutation and a mutation in NS4.

### NS4^V11I^ mutation is sufficient to rescue replication of MNV^CW3^ NS6^F182C^ and wild-type MNV independent of polyprotein processing

**Fig 2:**
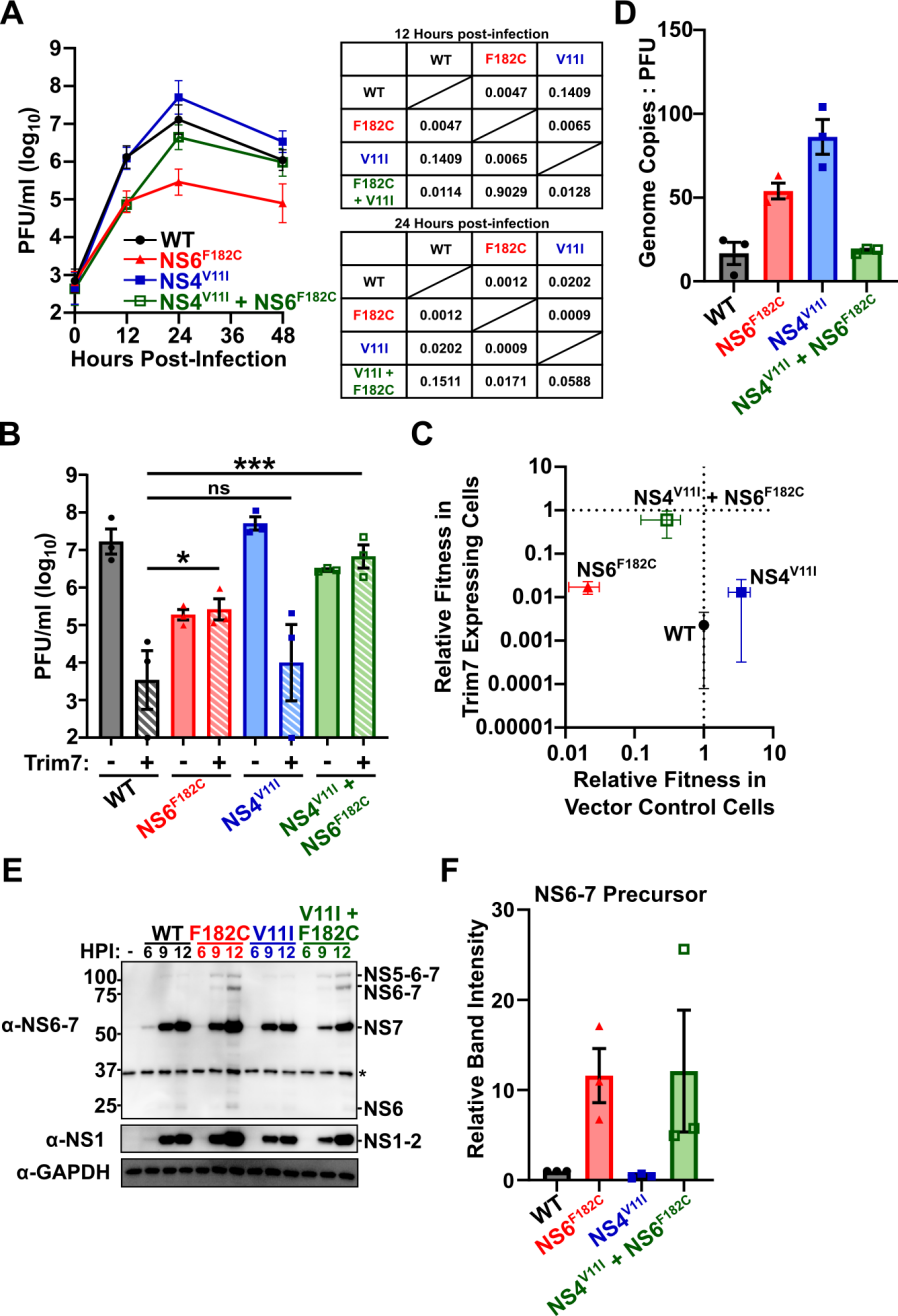
NS4^V11I^ mutation confers rescue of attenuated NS6^F182C^ replication independent of polyprotein processing. (**A**) Wild-type BV2 cells were infected with indicated MNV strains derived from MNV molecular clones at an MOI of 0.05. Viral titers were enumerated using plaque assays at the indicated time points. Data are shown as mean ± SEM from four independent experiments. Tables show *P*-values of statistical comparisons of viral titers at 12 h and 24 h post-infection. Two-way ANOVA with Tukey’s multiple comparisons test. (**B**) Vector control and Trim7-overexpressing BV2 cells were infected with indicated MNV strains at an MOI of 0.05. Viral titers were enumerated using plaque assays at 24 h post-infection. Data are shown as mean ± SEM from three independent experiments. Two-way ANOVA with Tukey’s multiple comparisons test. (**C**) Mean fitness of indicated MNV strains in vector control (*x*-axis) and Trim7-overexpressing BV2 cells (*y*-axis) 24 h post-infection. Data are relative to the replication of wild-type MNV in vector BV2 cells in each experiment. Dotted lines are drawn at one for both the *x*- and *y*-axis to represent the absolute fitness level of WT MNV in vector control cells as a reference. Data are shown as mean ± SEM from three independent experiments. (**D**) Genome copies-to-PFU ratios of indicated MNV strains, quantified by real-time quantitative PCR and plaque assay from indicated viral stocks. (**E**) Representative Western blot from wild-type BV2 cells infected with indicated MNV strains at an MOI of 5.0 and lysed at the indicated HPI. NS1/2, NS7, and NS6-7 precursor bands were identified by antibody-specific staining and expected molecular weights of the polyprotein precursors. The asterisk (*) indicates a non-specific band with the NS6-7 antibody. (**F**) Quantification of NS6-7 band intensities from Fig. 2E relative to total infection (NS1/GAPDH).

To confirm that NS4^V11I^ suppresses the growth defect of NS6^F182C^, we generated molecular clones containing both variants individually and in combination. The double mutant (NS4^V11I^ + NS6^F182C^) enhanced the overall replication of MNV compared to the NS6^F182C^ mutant alone in BV2 cells (Fig. 2A). However, this suppression of attenuation only happened at later time points (e.g., 24 h), as there was no difference in single-cycle replication between these two viruses (Fig. 2A). Interestingly, the addition of NS4^V11I^ to a non-attenuated (wild-type) virus had a similar enhancement effect compared to the wild-type virus (Fig. 2A). To test if the fitness advantage of the NS4^V11I^ + NS6^F182C^ double mutant is maintained in a Trim7-overexpressing environment, we examined growth properties of the MNV strains in BV2 cells expressing either a vector control or Trim7. Wild-type MNV is strongly inhibited by Trim7 (Fig. 2B and C) (14). A virus harboring the NS6^F182C^ mutation replicates to similar levels in Trim7 and vector control cells, albeit with less overall replication than the parental virus in wild-type cells (Fig. 2B and C). The NS4^V11I^ mutation on the wild-type MNV backbone does not confer a rescue of viral replication in Trim7 cells, while the double mutant NS4^V11I^ + NS6^F182C^ maintains Trim7 resistance and has enhanced replication in both vector control and Trim7 cells (Fig. 2B and C). Overall, the introduction of the NS4^V11I^ mutation rescues the antagonistic pleiotropy imposed by the NS6^F182C^ mutation as evidenced by its increase in overall fitness in all conditions tested (Fig. 2C). Interestingly, this increase in replication did not correlate with changes in the ratio of viral genomes to plaque-forming units (PFU) of our stocks (Fig. 2D), suggesting that the NS4^V11I^ viruses do not have a higher specific infectivity.

Position 182 falls within the 3C-protease cleavage site of NS6-7, and mutation of this site from phenylalanine to cysteine (F182C) diminishes, but does not abolish, the cleavage of NS6-7 (14). It is important to note that abolition of the NS6-7 protease cleavage site does not generate infectious MNV (19). Therefore, we tested whether NS4^V11I^ enhances NS6-7 processing, which could account for the rescue of viral replication. Consistent with previous findings, viruses containing the NS6^F182C^ mutation had an accumulation of NS6-7 polyprotein precursors, including NS6-7 and NS5-6-7, which was not apparent in the wild-type virus (Fig. 2E and F). The NS4^V11I^ + NS6^F182C^ double mutant had similar increases in NS6-7 precursor proteins detected during infection (Fig. 2E and F). In contrast, the NS6-7 polyprotein pattern of NS4^V11I^ was similar to that of wild-type MNV (Fig. 2E and F). Thus, these data indicate that NS4^V11I^ improves viral replication independently of polyprotein processing.

### NS4^V11I^ confers increased viral spread through enhanced infectivity after initial infection

**Fig 3:**
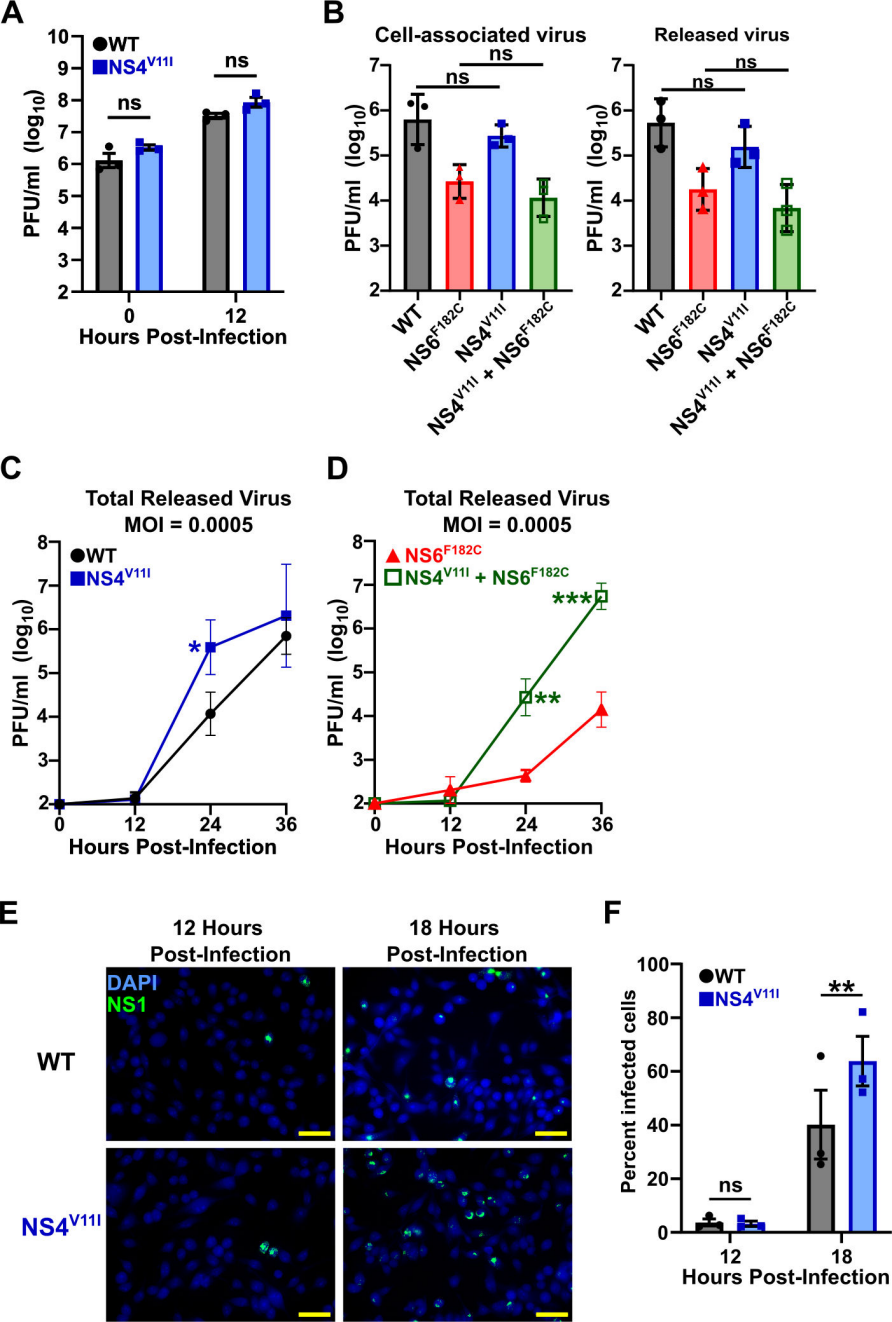
NS4^V11I^ promotes viral spread through increasing the number of MNV-infected cells after initial infection. (**A**) Wild-type BV2 cells were infected with wild-type MNV^CW3^ or MNV^CW3^ NS4^V11I^ at an MOI of 5.0, and viral titers were determined by plaque assay at indicated time points. Data are shown as mean ± SEM from three independent experiments. Two-way ANOVA with Sidak’s multiple comparisons test. (**B**) Wild-type BV2 cells were infected with indicated MNV strains at an MOI of 0.05. At 12 h post-infection, cell-associated (representing viral particles within cells) and released virus (representing viral particles in culture supernatants) were harvested. Viral titers were determined by plaque assay. Data are shown as mean ± SEM from three independent experiments. One-way ANOVA with Tukey’s multiple comparisons test. (**C and D**) Wild-type BV2 cells were infected with indicated viruses at an MOI of 0.0005. At indicated time points, supernatants were collected and viral titers enumerated via plaque assay. Data are shown as mean ± SEM from three independent experiments. One-way ANOVA with Tukey’s multiple comparisons test. ns, *P < 0.05, **P < 0.005, ***P < 0.0005. (**E**) Representative fluorescence micrographs of BV2 cells infected with wild-type MNV^CW3^ or MNV^CW3^ NS4^V11I^ at an MOI of 0.05. Cells were stained with DAPI (blue) and anti-NS1 (green) at either 12 or 18 h post-infection. Scale bar represents 50 µm. (**F**) Quantification of cells staining positive for NS1 as depicted in panel **E**. Data are shown as mean ± SEM from three independent experiments. One-way ANOVA with Tukey’s multiple comparisons test.

Since we observed an NS4^V11I^-mediated increase in viral replication at 24 h post-infection but not at 12 h post-infection (Fig. 2A), we further tested whether this mutation impacted single-cycle replication. We assessed viral replication at 12 h post-infection at a high MOI and found no difference in replication between wild-type and NS4^V11I^ (Fig. 3A). Additionally, there was no significant difference in levels of cell-associated virus and released virus at 12 h post-infection in the NS4^V11I^ viruses relative to their counterparts (Fig. 3B). To further confirm an increase in viral spread after initial infection, we utilized an infection model with 100-fold less virus than our typical low-MOI (0.05 vs 0.0005) and detected multiple-log increases in infectious viral release at later time points for viruses harboring the NS4^V11I^ mutation (Fig. 3C and D). To evaluate whether the increase in viral titers by NS4^V11I^ viruses is a result of infecting more cells over time, we quantified the number of cells infected using immunofluorescence staining. Infection levels are similar at 12 h post-infection in a single cycle of replication; however, NS4^V11I^ spread rapidly to infect more cells by 18 h post-infection (Fig. 3E and F). Taken together, our data indicate that NS4^V11I^ primarily enhances viral spread at later stages of infection by enabling more cells to be infected after infection has initiated.

### NS4^V11I^ spreads faster to neighboring cells in culture

**Fig 4:**
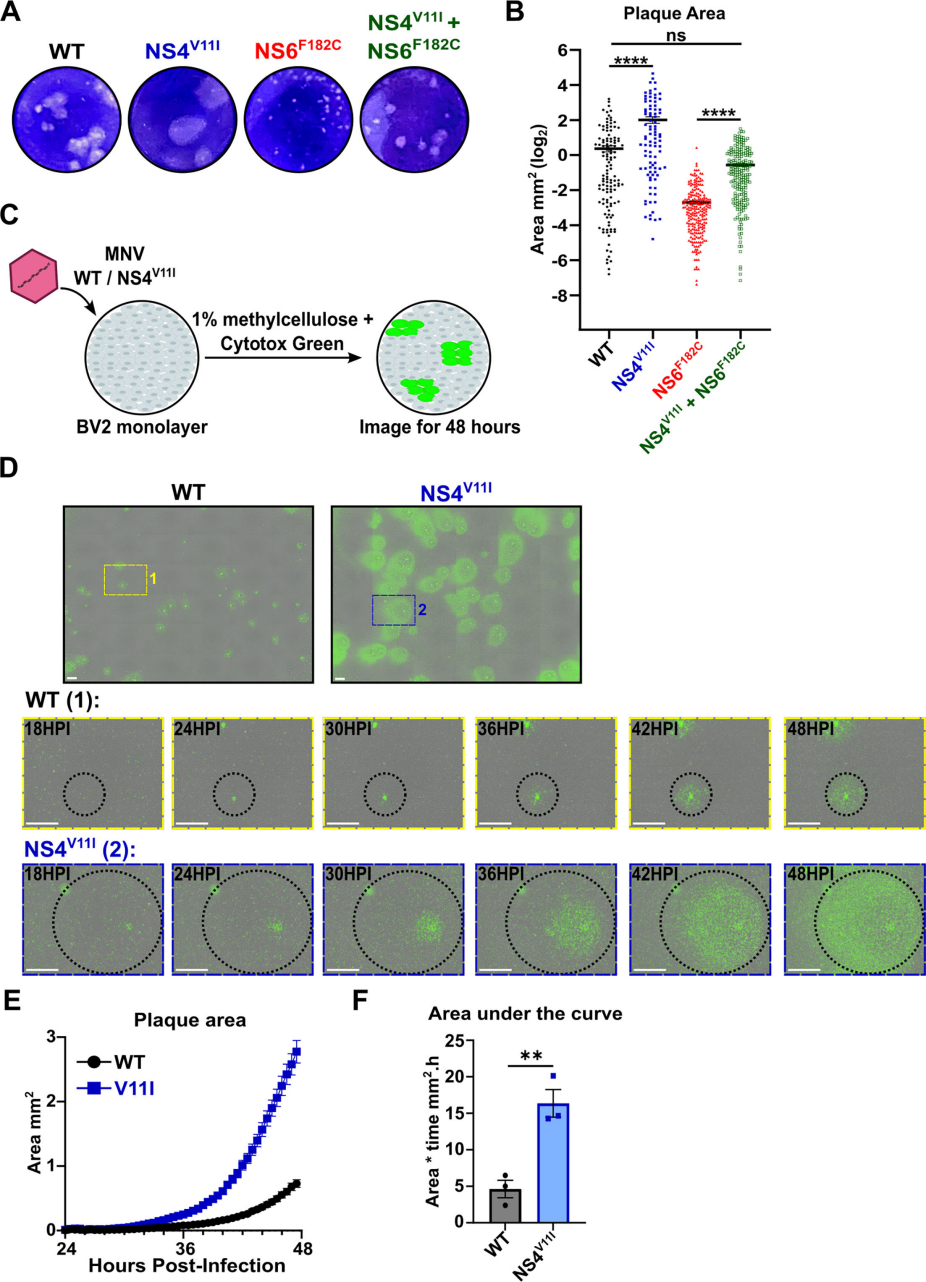
NS4^V11I^ leads to faster viral spread. (**A**) Representative plaque assay morphologies of wild-type BV2 cells infected with indicated viral strains. (**B**) Quantification of plaque sizes of infections in (**A**). Plaque areas were measured in ImageJ. Each point represents a single plaque. Data are shown as mean ± SEM from four independent experiments. ns, *P < 0.1, **P < 0.01, ***P < 0.001, ****P < 0.0001, Kruskal-Wallis test with Dunn’s multiple comparisons. (**C**) Schematic of experimental strategy for real-time imaging of MNV plaque growth. Wild-type BV2 cells were infected with MNV (~100 PFU per well) and incubated with 1% methylcellulose and 125 nM Cytotox Green dye. Phase and green-fluorescent images were taken every 2 h for the first 24 h post-infection and every 30 min for the next 24 h. (**D**) (Top) Representative stitched fluorescent microscopy image taken on IncuCyte at 48 h post-infection of wild-type BV2 cells infected as in panel **C**. Green dye represents apoptotic cells detected by Cytotox Green. (Bottom) Time-lapse fluorescence microscopy of selected single plaque growth of wild-type or NS4^V11I^ viral infection over 48 h post-infection. Scale bar represents 1 mm. (**E**) Quantification of plaque area from fluorescent microscopy from the IncuCyte images (**D**) for 48 h post-infection. Data represented as mean ± SEM from three independent experiments (*n* = 80–93 plaques per condition). (**F**) Area under the curve calculated individually from three independent experiments as in panel **E**. Data represented as mean ± SEM from three independent experiments. **P < 0.05, Welch’s *t*-test.

We were intrigued by the dramatic increase in viral replication of viruses containing either NS4^V11I^ or the combination of NS6^F182C^ and NS4^V11I^ in multicycle growth curves but not in any measurement of single-cycle replication (Fig. 3). Interestingly, during these experiments, we observed drastic differences in the size of plaques formed by these MNV strains (Fig. 4A). The specialist, attenuated NS6^F182C^ virus formed very small plaques, which were rescued to original plaque sizes in the double mutant NS4^V11I^ + NS6^F182C^ virus (Fig. 4A and B). NS4^V11I^ mutant alone also exhibited plaques much larger than wild-type virus (Fig. 4A). These data suggest an increase in viral spread when NS4^V11I^ mutation is present.

To quantify the kinetics of viral spread and plaque formation, we designed an assay for real-time measurement of MNV plaque formation (Fig. 4C). This assay incorporates a very low infection (100 plaque-forming units per well) with the viscous liquid overlay methylcellulose to limit viral diffusion. Additionally, we supplemented the media with Cytotox Green dye which marks dead cells and can be used as a surrogate for viral replication as there is no real-time live monitoring system for MNV replication. We imaged the cells over 48 h. We focused our efforts on characterizing the differences between wild-type MNV and MNV containing NS4^V11I^. These two viruses both produce robust, easily detectable plaques and demonstrate an NS4^V11I^-mediated enhancement of viral spread. Using Cytotox Green to mark plaques, we observe nearly a threefold difference in plaque size after 48 h, consistent with our crystal violet-based measurements (Fig. 4B, D, and E). Plaque initiation was detectable at 24 HPI for both wild-type and NS4^V11^-containing viruses (Fig. 4E). Despite the initiation event occurring at a similar time point, we observed a significant increase in the spread of the NS4^V11I^ viral plaques over time (Fig. 4D and E). Quantification of nearly 100 plaques in each condition across multiple experiments demonstrated that despite heterogeneity in plaque area within a sample, the NS4^V11I^ plaques were larger and spread faster (Fig. 4E). The area under the curves from three independent experiments was significantly greater in NS4^V11I^ compared to wild-type infection, demonstrating reproducibility across independent experiments (Fig. 4F). Taken together, these data demonstrate that the NS4^V11I^-containing viruses are able to spread to neighboring cells significantly faster and likely account for the suppression of the NS6^F182C^ attenuating phenotype.

### Rapid MNV^CW3^ NS4^V11I^ viral spread is not due to modulation of interferon signaling

**Fig 5:**
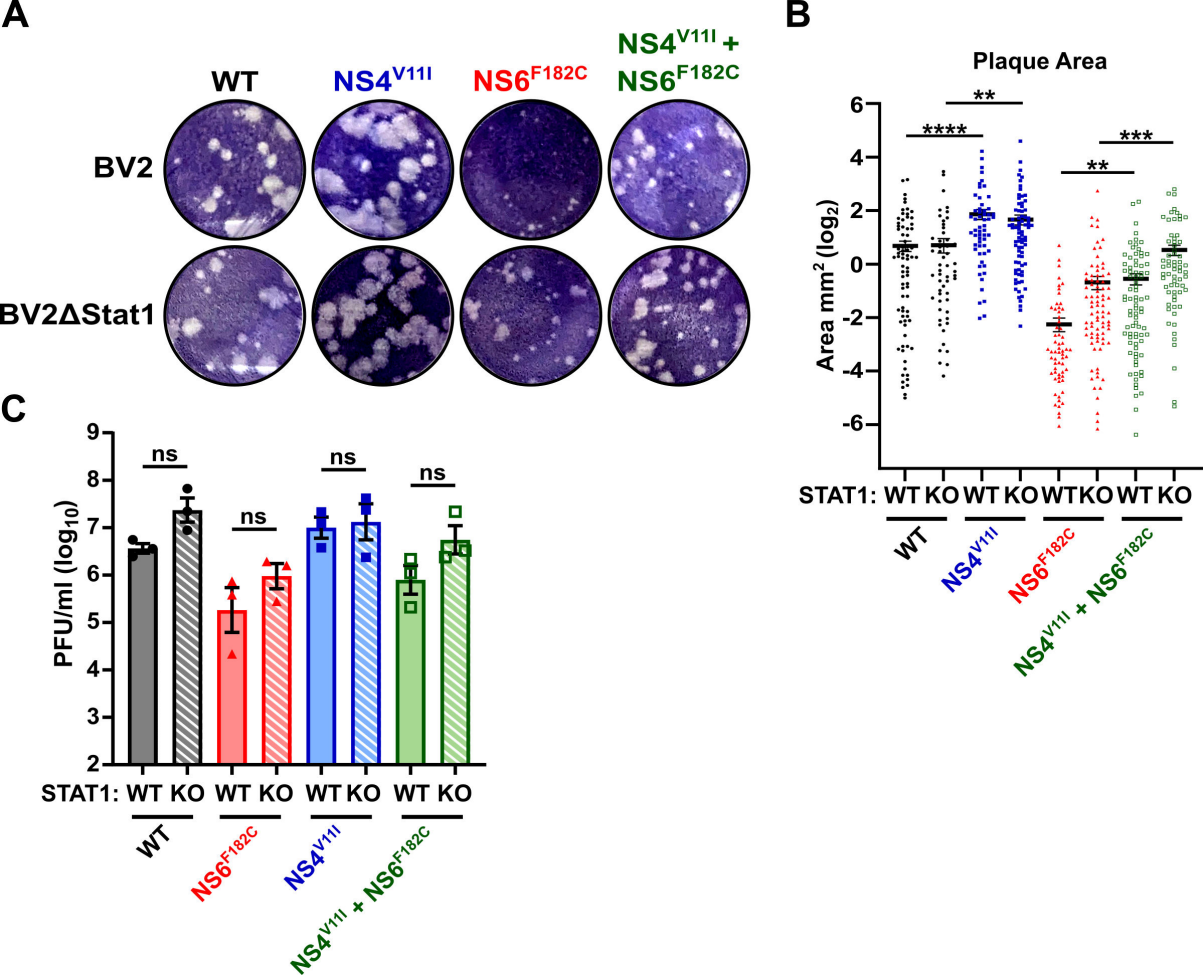
Rapid spread of MNV^CW3^ NS4^V11I^ is not due to modulation of interferon signaling. (**A**) Representative plaque assay morphologies of wild-type BV2 (top) and BV2ΔSTAT1 (bottom) infected with indicated MNV strains. (**B**) Quantification of plaque sizes of infections in (**A**). Plaque areas were measured in ImageJ. Each point represents a single plaque. Data are shown as mean ± SEM from four independent experiments. ns, *P < 0.1, **P < 0.01, ***P < 0.001, ****P < 0.0001, Kruskal-Wallis test with Dunn’s multiple comparisons. (**C**) Wild-type and ΔSTAT1 BV2 cells were infected with indicated MNV strains at an MOI of 0.05. Viral titers were enumerated using plaque assays at 24 h post-infection. Data are shown as mean ± SEM from three independent experiments. Two-way ANOVA with Tukey’s multiple comparisons test.

Since the NS4^V11I^ mutation enables a more rapid spread of infection from a single infected cell to its neighboring cells, we hypothesized that NS4^V11I^ may be modifying host defense pathways to infect neighboring cells more easily. Type I and type III IFNs are a major innate immune defense against viruses, including norovirus (20–23). To determine whether the NS4^V11I^ mutation improves viral spread to neighboring cells due to modulation of interferon signaling, we compared the plaque size of viruses grown on wild-type BV2 or BV2ΔSTAT1, which lack the ability to respond to all IFNs. There was no difference in the plaque morphology or size of wild-type MNV in wild-type or BV2ΔSTAT1 cells (Fig. 5A and B). We observed similar trends in the viruses containing the NS4^V11I^ mutation (Fig. 5A and B). Interestingly, viruses that contained the NS6^F182C^ mutation had an increase in plaque size when grown on BV2ΔSTAT1 cells compared to wild-type BV2 cells (Fig. 5A and B). Importantly, the NS4^V11I^ increase in viral spread as measured by plaque size occurred independently of STAT1-dependent signaling (Fig. 5A and B). For example, the NS4^V11I^ and NS6^F182C^ double mutant had an increase in plaque size compared to NS6^F182C^ single mutant in both wild-type and BV2ΔSTAT1 cells (Fig. 5A and B). Infectious particle production at 24 h post-infection showed similar trends as those of the plaque size; however, these data did not reach statistical significance at this time point (Fig. 5C). These data demonstrate that the increase in viral spread by NS4^V11I^ does not occur via modulation of the IFN pathway.

## DISCUSSION

Viruses are a powerful genetic tool to study evolution due to their compact genome and prolific replication within a relatively short time frame. These traits enable viruses to rapidly adapt to new environments. Studying these adaptations has enabled the development of live attenuated vaccines, modeling of zoonotic transmission, discovery of immune barriers, and identification of antiviral targets (1, 2, 24). In many cases, while these adaptations broaden the host range, there is a loss of general replication in the original settings. While genetic reversion can allow these viruses to recover their fitness cost, there is less information about how suppressive mutations overcome antagonistic pleiotropy. Here, using an unbiased forward genetic screen, we describe such a situation in which a suppressive mutation is acquired to restore the replication of an adaptive, attenuated MNV strain to the levels of its original precursor. MNV adapted to Trim7-overexpressing cells (MNV NS6^F182C^) can no longer grow robustly in wild-type or other general settings (14). However, the acquisition of a mutation in NS4 (valine-to-isoleucine at position 11; V11I) converts the attenuated, specialist virus to a robustly replicating virus (Fig. 1 and 2). These findings have several implications on norovirus biology and viral evolution.

Viruses within the Caliciviridae family differ in their polyprotein processing of NS6 and NS7 (also known as Pro-Pol) (25, 26). Norovirus is unique among caliciviruses for its separation of NS6 and NS7, and surprisingly, this cleavage event is required for recovery of infectious virus (19). In support of this notion, the NS6^F182C^ mutation, which has decreased cleavage of the NS6-7 precursor, is positively selected as a means of resisting Trim7 expression, yet is highly attenuated in native settings (14). It remains unclear why the cleavage of NS6-7 is uniquely required in noroviruses. Our data suggest one mechanism may be that cleaved norovirus NS6 is more efficient at antagonizing innate immunity and is necessary for efficient viral spread. In support of this model, we find that a single amino acid mutation in MNV NS4 (NS4^V11I^) is sufficient to rescue the attenuated replication of NS6^F182C^. This mutation did not alter the defective polyprotein processing of NS6^F182C^ but rather increased the spread of both the parental virus and the NS6^F182C^ attenuated virus (Fig. 2). Increased viral spread via NS4^V11I^ may compensate for a decrease in NS6-mediated innate immune antagonism. In support of this hypothesis, NS6 has several reported immune antagonism functions including alteration of host translation through cleavage of poly(A)-binding protein (27), and cleavage of NF-κB essential modulator protein (28). Additionally, other critical immune nodes are targeted by viral 3C-proteases, including by related caliciviruses (29). However, it is also possible that NS6^F182C^ has multiple bottlenecks in viral replication, and the NS4^V11I^ is a simple method to significantly boost viral spread and increase fitness.

The function of NS4 during norovirus replication is still unknown. Based on positional homology in the genome, NS4 is a predicted “3A-like” protein similar to picornavirus 3A protein. 3A proteins have diverse functions but can be largely classified as membrane remodelers and immune antagonists. For example, poliovirus 3A regulates membrane remodeling in host cells (30) and inhibits cellular protein secretory pathways to decrease inflammatory immune response (31, 32). Indeed, ectopic expression studies of human and mouse norovirus NS4 proteins have demonstrated a propensity for membrane rearrangement (33), possible alterations in the general secretory pathway (34), and the potential for the activation of innate immunity through sensing cytosolic DNA (35). However, the physiological role of NS4 during infection remains enigmatic. It is possible that the comparison of the valine-to-isoleucine switch in NS4 during infection may provide novel insights into the mechanism by which norovirus utilizes NS4. Future studies may leverage the significant difference in plaque sizes of NS4 variants as a robust platform to dissect the host and viral pathways enabling increased viral spread.

Position 11 of NS4 among MNV strains is predominantly valine, although a significant number of strains have an isoleucine at this position. Our *in vitro* data demonstrate that NS4^V11I^ replicates to higher titers and spreads better *in vitro*, yet a previous study found no difference in pathogenesis in STAT1^-/-^ mice infected with either variant (18). It remains unclear what evolutionary pressures differ *in vitro* and *in vivo* to have such differences in selection and maintenance of these different variants. Valine and isoleucine are nearly identical chemically, yet the viral spread of NS4^V11I^ is dramatic and robust (Fig. 4). Future work investigating the molecular basis for these different phenotypes will likely increase our understanding of norovirus evolution and the function of NS4.

## MATERIALS AND METHODS

### Cell culture

BV2, BV2-ΔSTAT1 (Dr. Skip Virgin, Washington University; [36]), and HEK-293T cells (ATCC) were cultured in Dulbecco’s Modified Eagle Medium supplemented with 5% fetal bovine serum. Trim7-expressing stable cell lines were generated by lentiviral transduction. Briefly, pCDH-MCS-T2A-Puro-MSCV-Trim7 isoform 1 (13), lentiviral packing vector (psPax2), and pseudotyping vector (pCMV-VSV-G) were co-transfected into HEK-293T cells using OptiMEM (Sigma) and Transit-LT1 (Mirus). Lentivirus was collected 48 h post-transfection, filtered through a 0.45 µm filter, and added to cells. Forty-eight hours post-transduction, cells were selected with 1 µg/mL puromycin.

### MNV assays

MNV^CW3^ stocks were generated from plasmids encoding the complete MNV^CW3^ genome (GenBank ID EF014462.1), purified and titered by plaque assay as described previously (37, 38). NS4^V11I^ and NS6^F182C^ mutations were introduced through splicing by overlap extension PCR in MNV molecular clones. All plasmids were verified through sequencing prior to use. Mutant viruses were generated on the plasmid containing the MNV^CW3^ backbone similar to parental MNV^CW3^ stocks. Genetic identity of viral stocks was confirmed by sequencing viral RNA. MNV replication assays were performed as described previously (13) with the indicated multiplicity of infection (MOI 0.0005, 0.05, or MOI 5).

MNV protease processing assay was performed by infecting wild-type BV2 cells at an MOI of 5. Cells were lysed at indicated times post-infection in Laemmli buffer (Bio-Rad). Lysates were resolved via SDS-PAGE, transferred to polyvinylidene difluoride (PVDF) membranes, and probed with the indicated antibodies.

For quantification of viral genomes, RNA was isolated from 50 to 100 µL of indicated viral P1 stocks using the Direct-zol RNA MiniPrep Kit (Zymo Research) following the manufacturer’s protocol. Purified RNA was used for cDNA synthesis using the M-MLV Reverse Transcriptase (Invitrogen). TaqMan quantitative PCR (qPCR) for MNV was performed in triplicate on each sample and standard with forward primer 5′-GTGCGCAACACAGAGAAACG-3′, reverse primer 5′-CGGGCTGAGCTTCCTGC-3′, and probe 5′−6FAM-TAGTGTCTCCTTTGGAGCACCTA-BHQ1-3′.

To determine the cell-associated and released virus quantities, wild-type BV2 cells were seeded at 2.5 × 10^5^ per well in a 24-well plate or 5 × 10^4^ per well in a 96-well plate. Cells were allowed to adhere overnight and infected with MNV^CW3^ wild-type and mutant viruses at an MOI of 0.05 or 0.0005. At indicated time points, supernatants were collected from the wells, and fresh media was added back to the wells. All samples were frozen at −80°C. Viral titers were determined by plaque assay.

MNV replication complex formation was visualized by immunofluorescence staining of MNV NS1 in BV2 cells infected with MNV^CW3^ wild-type and NS4^V11I^ viruses at an MOI of 0.05 on coverslips. Cells were fixed in paraformaldehyde (Santa Cruz) at 12 and 18 h post-infection and stained for NS1 and DAPI (nuclear staining).

Viral spread plaque assays were performed by seeding wild-type BV2 or BV2ΔSTAT1 at 2.5 × 10^5^ cells per well in a 24-well plate. After adhering overnight, cells were infected with a serial dilution of 10, 100, and 1000 PFU of the indicated viruses. After 1 h of incubation, the virus was aspirated, and 1% methylcellulose was added to the wells. Cells were stained with crystal violet at 48 h post-infection, and stained plates were imaged. Quantification of plaque sizes was performed in ImageJ.

### Viral evolution

MNV passaging experiments were conducted in BV2-Trim7 cells similar to our previous study (14). Briefly, BV2-Trim7 cells were seeded at 1 × 10^7^ in a 10 cm^2^ plate. After adhering overnight, attenuated parental MNV^CW3^ NS6^F182C^ (P0) was infected at MOI 5. Twenty-four hours post-infection, virus was harvested and clarified from the supernatant to obtain MNV^CW3^ NS6^F182C^ P1. One milliliter of the P1 virus was added to BV2-Trim7 cells in a 10 cm^2^ plate. Subsequent passaging of the virus was done at 48 h post-infection up to P6. After passage 6, viral passaging was continued at every 12 h post-infection up to P14. This provided greater evolutionary selective pressure on the virus. One milliliter of the final clarified P14 viral supernatant was used to isolate total RNA using Zymo Direct-zol kit. cDNA was synthesized using the M-MLV Reverse Transcriptase kit (Invitrogen) following manufacturer’s protocols. Fragment PCR was performed on the viral cDNA using primers spanning the MNV genome. The PCR products were then purified and sequenced by Sanger sequencing.

### Antibodies, Western blotting, and immunofluorescence

Rabbit polyclonal anti-NS6-7 (a kind gift from Kim Green), mouse monoclonal anti-NS1 (a kind gift from Sanghyun Lee), mouse anti-GAPDH HRP (Sigma-Aldrich), anti-rabbit IgG HRP (Sigma-Aldrich), and anti-mouse IgG (Sigma-Aldrich) were used for Western blotting. Goat anti-mouse IgG (H+L), Alexa Fluor 488 (Thermo Fisher), and DAPI (Thermo Fisher) were used for immunofluorescence staining. Western blot band intensities and NS1 complexes were quantified using ImageJ.

### IncuCyte-based real-time quantification of MNV plaques

Wild-type BV2 cells were seeded at 2 × 10^6^ per well in a six-well plate. After adhering overnight, cells were infected with MNV^CW3^ wild-type or MNV^CW3^ NS4^V11I^ at 100 PFU per well. After 1 h of incubation, the virus was aspirated from the wells. One percent methylcellulose supplemented with 125 nM Cytotox Green dye (Sartorius) was added to the wells. The methylcellulose was first spun down at 4,000 rpm for 10 min to remove any particulates that may interfere with imaging. The plate was placed in the IncuCyte, and images were acquired in the phase and green channels. Thirty-six images were taken per well at 4× magnification at every 2 h up to 24 h post-infection and every 30 min for the next 24 h. Timelapse videos of each image section were exported and stitched together using Adobe Premiere Pro. Videos were processed on ImageJ, and plaque area of selected plaques was calculated over time for each experiment.

## Data Availability

All relevant data are contained within the manuscript.
